# UNPACKING THE RELATIONSHIP BETWEEN EDUCATION AND COGNITIVE DECLINE AND DEMENTIA

**DOI:** 10.1093/geroni/igae098.0274

**Published:** 2024-12-31

**Authors:** Pamela Herd, Katrina Walsemann

**Affiliations:** Chair; Co-Chair

## Abstract

This session, which brings together a set of papers from participants in the Network on Education, Biosocial Pathways, and Dementia in Diverse Populations (EdDem), attends to the relationship between education and cognitive function and dementia in later life. The papers in this session explore two major themes. First, they examine the role of early and mid-life influences on these relationships. How do exposures to poor health in early life influence both educational attainment and subsequent risk for poor cognitive outcomes in later life? How does the content of early life schooling, including the rigor of the curriculum, influence both educational attainment and subsequent risk for poor cognitive outcomes in later life? The papers also consider the role of midlife factors, including occupation, health behaviors, and health conditions, in mediating the relationship between education and later life cognitive outcomes. Finally, these papers explore how the relationship between education and later life cognition varies within and across middle- and high-income countries. Overall, this set of papers helps unpack the persistent and robust relationship between education and cognitive function and dementia in later life. Session attendees will also have the opportunity to learn about the NIH-funded EdDem network, including funding and network related opportunities.

